# Crystal structure of disilver(I) dizinc(II) iron(III) tris­(orthovanadate) with an alluaudite-type structure

**DOI:** 10.1107/S205698901801071X

**Published:** 2018-07-27

**Authors:** Nour El Houda Lamsakhar, Mohammed Zriouil, Abderrazzak Assani, Mohamed Saadi, Lahcen El Ammari

**Affiliations:** a Laboratoire de Chimie Appliquée des Matériaux, Centre des Sciences des Matériaux, Faculty of Sciences, Mohammed V University in Rabat, Avenue Ibn Batouta, BP 1014, Rabat, Morocco

**Keywords:** crystal structure, transition metal vanadate, solid-state reaction, alluaudite structure type., crystal structure

## Abstract

The title compound, Ag_2_Zn_2_Fe(VO_4_)_3_, has been synthesized by solid-state reactions and belongs to the alluaudite structure family. In the crystal structure, four sites are positioned at special positions. One silver site is located on an inversion centre (Wyckoff position 4*b*), and an additional silver site, as well as one zinc and one vanadium site, on twofold rotation axes (4*e*). One site on a general position is statistically occupied by Fe^III^ and Zn^II^ cations that are octa­hedrally surrounded by O atoms. The three-dimensional framework structure of the title vanadate results from [(Zn,Fe)_2_O_10_] units of edge-sharing [(Zn,Fe)O_6_] octa­hedra that alternate with [ZnO_6_] octa­hedra so as to form infinite chains parallel to [10]. These chains are linked through VO_4_ tetra­hedra by sharing vertices, giving rise to layers extending parallel to (010). Such layers are shared by common vanadate tetra­hedra. The resulting three-dimensional framework delimits two types of channels parallel to [001] in which the silver sites are located with four- and sixfold coordination by oxygen.

## Abstract 2

The transition metal orthovanadate, Ag_2_Zn_2_Fe(VO_4_)_3_, crystallizes in an alluaudite-type structure. Zn^II^ and Fe^III^ atoms are statistically occupied on one general site.

## Chemical context   

The crystal structure of the mineral alluaudite with general formula *A*(1)*A*(2)*M*(1)*M*(2)_2_(*X*O_4_)_3_ was determined nearly fifty years ago by Moore (1971 ▸). In the structure, the two *A* sites can be occupied by mono- or divalent cations of medium size, and the *M*(1) and *M*(2) sites can accommodate di- or trivalent cations, which are generally transition metals and are octa­hedrally surrounded. The specific feature of the alluaudite structure is the existence of two channels parallel to [001] in which the *A*-site cations are located. As a result, alluaudite-type compounds can exhibit electronic and/or ionic conductivity (Hatert, 2008 ▸). In addition, alluaudite-type compounds have been reported as materials for fossil energy conversion, as sensor materials and storage energy materials (Korzenski *et al.*, 1998 ▸), and as materials used in catalysis (Kacimi *et al.*, 2005 ▸).

Accordingly, the synthesis and structural characterization of new alluaudite-type phosphates and vanadates within pseudo-ternary *A*_2_O/*M*O/P_2_O_5_ or pseudo-quaternary *A*_2_O/*M*O/Fe_2_O_3_/P_2_O_5_ systems using hydro­thermal or solid-state reactions was the focus of our current research. Obtained phases are, for example, NaMg_3_(HPO_4_)_2_(PO_4_) (Ould Saleck *et al.*, 2015 ▸), Na_2_Co_2_Fe(PO_4_)_3_ (Bouraima *et al.*, 2015 ▸) or Na_1.67_Zn_1.67_Fe_1.33_(PO_4_)_3_ (Khmiyas *et al.*, 2015 ▸). We have also succeeded in preparing the first vanadate-based alluaudite-type phase (Na_0.70_)(Na_0.70_,Mn_0.30_)(Fe^III^,Fe^II^)_2_Fe^II^(VO_4_)_3_ (Benhsina *et al.*, 2016 ▸). A second alluaudite-type vanadate with composition Na_2_(Fe^III^/Co^II^)_2_Co^II^(VO_4_)_3_ was prepared by Hadouchi *et al.* (2016 ▸) shortly afterwards.

In this context, the current exploration of *A*_2_O/*M*O/Fe_2_O_3_/V_2_O_5_ systems, where *A* is a monovalent cation and *M* a divalent cation, led to another vandanate with alluaudite-type structure, namely Ag_2_Zn_2_Fe(VO_4_)_3_. Its synthesis and crystal structure are reported in this article.

## Structural commentary   

**Figure 1:**
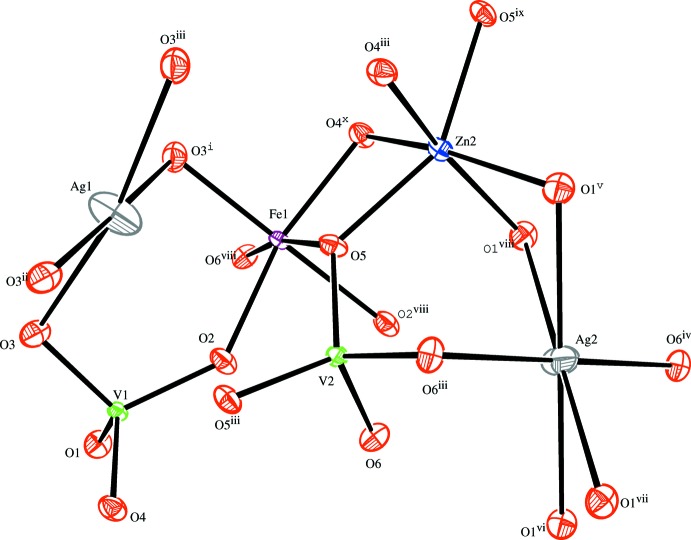
The principal building units in the structure of the title compound. Displacement ellipsoids are drawn at the 50% probability level. [Symmetry codes: (i) *x*, −*y* + 1, *z* − 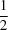; (ii) −*x* + 1, −*y* + 1, −*z* + 2; (iii) −*x* + 1, *y*, −*z* + 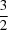; (iv) *x*, −*y*, *z* − 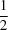; (v) *x* + 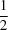, −*y* + 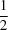, *z* − 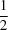; (vi) −*x* + 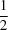, *y* − 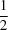, −*z* + 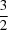; (vii) *x* + 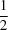, *y* − 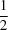, *z*; (viii) −*x* + 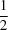, −*y* + 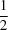, −*z* + 1; (ix) −*x* + 1, *y*, −*z* + 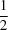; (*x*) *x*, *y*, *z* − 1.]

The principal building units of the crystal structure of the new member of the alluaudite-type family are represented in Fig. 1 ▸. All atoms are in general positions except for four atoms that are located on special positions. Ag1 is located on an inversion centre (Wyckoff position 4*b*), and Ag2 as well as Zn2 and V2 are located on twofold rotation axes (4*e*) of space group *C*2/*c*. The *M*2 site is in a general position (8*f*) and statistically occupied by Fe1 and Zn1 atoms that are octa­hedrally surrounded by O atoms. Such a partial cationic disorder was also reported for the cobalt homologue Na_2_(Fe^III^/Co^II^)_2_Co^II^(VO_4_)_3_ (Hadouchi *et al.*, 2016 ▸).

**Figure 2:**
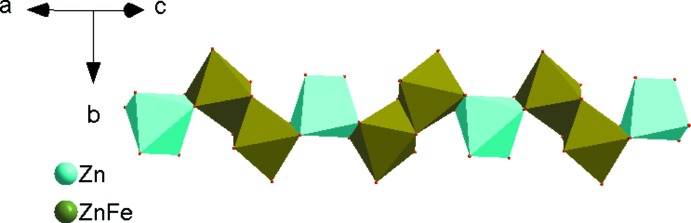
Edge-sharing [(Zn,Fe)1O_6_] and [Zn2O_6_] octa­hedra forming a kinked chain running parallel to [10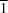].

**Figure 3:**
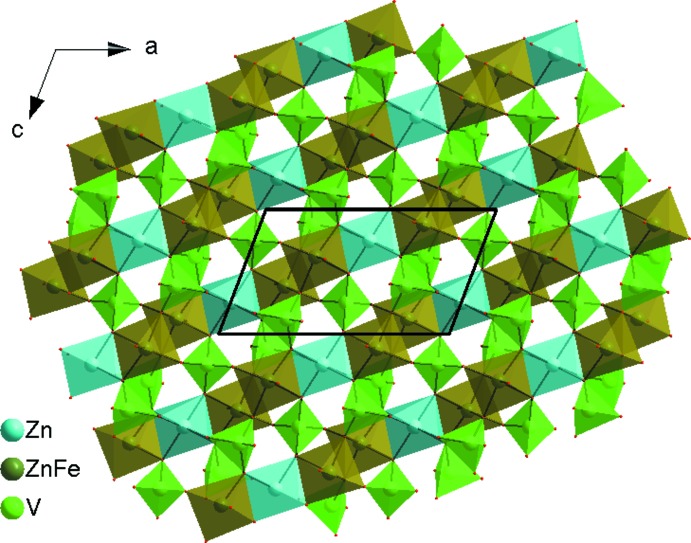
A layer perpendicular to (010), resulting from the connection of chains *via* the vertices of VO_4_ tetra­hedra and [ZnO_6_] octa­hedra.

**Figure 4:**
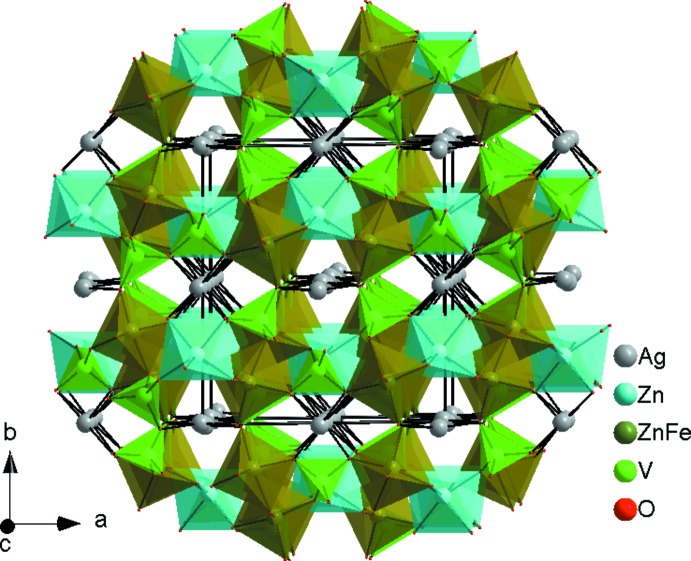
Polyhedral representation of Ag_2_Zn_2_Fe(VO_4_)_3_ showing the channels running parallel to the [001] direction.

The crystal structure of Ag_2_Zn_2_Fe(VO_4_)_3_ is made up from [(Zn,Fe)1_2_O_10_] dimers, resulting from edge-sharing [(Zn,Fe)1O_6_] octa­hedra, that are connected by a common edge to [Zn2O_6_] octa­hedra. The linkage of alternating [(Zn,Fe)1_2_O_10_] and [Zn2O_6_] units leads to infinite zigzag chains along [10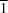] (Fig. 2 ▸). These chains are linked *via* the vertices of VO_4_ tetra­hedra into layers parallel to (010), as shown in Fig. 3 ▸. Adjacent layers are linked by V1O_4_ tetra­hedra into a three-dimensional framework structure that delimits two types of channels in which the Ag^I^ cations reside (Fig. 4 ▸). The Ag1 site is located in one channel and is surrounded by four oxygen atoms, whereas the Ag2 site in the second channel is surrounded by six oxygen atoms.

The calculated bond-valences sums (Brown & Altermatt, 1985 ▸) of the atoms in the structure are in the expected ranges for Ag^I^, Zn^II^, Fe^III^ and V^V^ and are as follows (values in valence units): Ag1 (0.83), Ag2 (1.11), Zn1 (1.95), Zn2 (2.20), Fe1 (2.67), V1 (4.98) and V2 (4.93); values of oxygen atoms range between 1.90 and 2.01 valence units.

## Database Survey   

Over the last twenty years, many synthetic alluaudite-type phosphates, arsenates, sulfates and molybdates have been reported, such as NaMnFe_2_(PO_4_)_3_ used as the positive electrode in sodium and lithium batteries (Trad *et al.*, 2010 ▸; Kim *et al.*, 2014 ▸; Huang *et al.*, 2015 ▸), Na_2.44_Mn_1.79_(SO_4_)_3_ used as a potential high-voltage cathode material (*ca* 4.4 V) for sodium batteries (Dwibedi *et al.*, 2015 ▸), K_0.13_Na_3.87_Mg(MoO_4_)_3_ as a promising compound for developing new materials with high ionic conductivity (Ennajeh *et al.*, 2015 ▸), or NaZn_3_(AsO_4_)(AsO_3_OH)_2_ (Đorđević *et al.*, 2015 ▸).

## Synthesis and crystallization   

Ag_2_Zn_2_Fe(VO_4_)_3_ was prepared by a solid-state reaction. A stoichiometric amount of silver nitrate (AgNO_3_), zinc acetate (Zn(CH_3_COO)_2_·2H_2_O), iron nitrate (Fe(NO_3_)_3_)·9H_2_O) and vanadium oxide (V_2_O_5_) was employed in the molar ratio Ag: Zn:Fe:V = 2:2:1:3 and put into a platinum cruicible. After different heat treatments at lower temperatures to remove water and other voliatile gaseous products, the reaction mixture was melted at 1033 K for 30 minutes, followed by slow cooling with a 5 K h^−1^ rate to room temperature. The resulting product contained parallelepipedic orange crystals corres­ponding to the studied title vanadate. In addition, small block-like crystals with poor quality and unidentified by X-ray powder diffraction were present.

## Refinement   

Crystal data, data collection and structure refinement details are summarized in Table 1 ▸. The remaining maximum and minimum electron density peaks in the final Fourier map are 0.40 Å away from Fe1 and 0.62 Å from Ag1, respectively. Due to charge neutrality, sites Zn1 and Fe2 were modelled as statistically occupied, assuming a trivalent oxidation state for the iron site.

## Supplementary Material

Crystal structure: contains datablock(s) I. DOI: 10.1107/S205698901801071X/wm5454sup1.cif

Structure factors: contains datablock(s) I. DOI: 10.1107/S205698901801071X/wm5454Isup2.hkl

CCDC reference: 1857879

Additional supporting information:  crystallographic information; 3D view; checkCIF report

## Figures and Tables

**Table 1:** Experimental details

Crystal data	
Chemical formula	Ag_2_Zn_2_Fe(VO_4_)_3_
*M*_r_	747.15
Crystal system, space group	Monoclinic, *C*2/*c*
Temperature (K)	296
*a*, *b*, *c* (Å)	11.8025 (2), 12.9133 (2), 6.8000 (1)
β (°)	110.759 (1)
*V* (Å^3^)	969.10 (3)
*Z*	4
Radiation type	Mo *K*α
μ (mm^−1^)	13.09
Crystal size (mm)	0.31 × 0.26 × 0.20

Data collection	
Diffractometer	Bruker X8 *APEX*
Absorption correction	Multi-scan (*SADABS*; Krause *et al.*, 2015 ▸)
*T*_min_, *T*_max_	0.596, 0.748
No. of measured, independent and observed [*I* > 2σ(*I*)] reflections	30791, 2662, 2437
*R*_int_	0.042
(sin θ/λ)_max_ (Å^−1^)	0.869

Refinement	
*R*[*F* ^2^ > 2σ(*F* ^2^)], *wR*(*F* ^2^), *S*	0.021, 0.048, 1.13
No. of reflections	2662
No. of parameters	95
Δρ_max_, Δρ_min_ (e Å^−3^)	1.36, −2.41

Computer programs: *APEX2* and *SAINT* (Bruker, 2014 ▸), *SHELXT2014* (Sheldrick, 2015*a* ▸), *SHELXL2014* (Sheldrick, 2015*b* ▸), *ORTEP-3 for Windows* (Farrugia, 2012 ▸), *DIAMOND* (Brandenburg, 2006 ▸) and *publCIF* (Westrip, 2010 ▸).

## supplementary crystallographic information

### Crystal data

**Table:**

Ag_2_Zn_2_Fe(VO_4_)_3_	*F*(000) = 1380
*M**_r_* = 747.15	*D*_x_ = 5.121 Mg m^−^^3^
Monoclinic, *C*2/*c*	Mo *K*α radiation, λ = 0.71073 Å
*a* = 11.8025 (2) Å	Cell parameters from 2662 reflections
*b* = 12.9133 (2) Å	θ = 2.4–38.1°
*c* = 6.8000 (1) Å	µ = 13.09 mm^−^^1^
β = 110.759 (1)°	*T* = 296 K
*V* = 969.10 (3) Å^3^	Parallelepiped, orange
*Z* = 4	0.31 × 0.26 × 0.20 mm

### Data collection

**Table:**

Bruker X8 APEX diffractometer	2662 independent reflections
Radiation source: fine-focus sealed tube	2437 reflections with *I* > 2σ(*I*)
Graphite monochromator	*R*_int_ = 0.042
φ and ω scans	θ_max_ = 38.1°, θ_min_ = 2.4°
Absorption correction: multi-scan (SADABS; Krause *et al.*, 2015)	*h* = −18→20
*T*_min_ = 0.596, *T*_max_ = 0.748	*k* = −22→22
30791 measured reflections	*l* = −11→9

### Refinement

**Table:**

Refinement on *F*^2^	0 restraints
Least-squares matrix: full	*w* = 1/[σ^2^(*F*_o_^2^) + (0.0126*P*)^2^ + 4.2342*P*] where *P* = (*F*_o_^2^ + 2*F*_c_^2^)/3
*R*[*F*^2^ > 2σ(*F*^2^)] = 0.021	(Δ/σ)_max_ = 0.001
*wR*(*F*^2^) = 0.048	Δρ_max_ = 1.36 e Å^−^^3^
*S* = 1.13	Δρ_min_ = −2.41 e Å^−^^3^
2662 reflections	Extinction correction: SHELXL2016 (Sheldrick, 2015*b*), Fc^*^=kFc[1+0.001xFc^2^λ^3^/sin(2θ)]^-1/4^
95 parameters	Extinction coefficient: 0.00163 (6)

### Special details

**Table:**

Geometry. All esds (except the esd in the dihedral angle between two l.s. planes) are estimated using the full covariance matrix. The cell esds are taken into account individually in the estimation of esds in distances, angles and torsion angles; correlations between esds in cell parameters are only used when they are defined by crystal symmetry. An approximate (isotropic) treatment of cell esds is used for estimating esds involving l.s. planes.

### Fractional atomic coordinates and isotropic or equivalent isotropic displacement parameters (Å^2^)

**Table:**

	*x*	*y*	*z*	*U*_iso_*/*U*_eq_	Occ. (<1)
Ag1	0.500000	0.49090 (3)	0.750000	0.02736 (7)	
Ag2	0.500000	0.000000	0.500000	0.02115 (6)	
Zn2	0.500000	0.23529 (2)	0.250000	0.00945 (6)	
Zn1	0.29222 (2)	0.34062 (2)	0.38041 (3)	0.00652 (5)	0.5
Fe1	0.29222 (2)	0.34062 (2)	0.38041 (3)	0.00652 (5)	0.5
V1	0.27045 (3)	0.38683 (2)	0.88206 (4)	0.00612 (5)	
V2	0.500000	0.20643 (3)	0.750000	0.00602 (6)	
O1	0.12116 (12)	0.39616 (11)	0.8338 (2)	0.0128 (2)	
O2	0.28524 (13)	0.31700 (11)	0.6746 (2)	0.0124 (2)	
O3	0.33803 (14)	0.50767 (11)	0.8997 (2)	0.0139 (2)	
O4	0.33926 (12)	0.32576 (11)	1.1233 (2)	0.0112 (2)	
O5	0.46319 (12)	0.27705 (11)	0.5152 (2)	0.0099 (2)	
O6	0.38484 (12)	0.12416 (10)	0.7343 (2)	0.0115 (2)	

### Atomic displacement parameters (Å^2^)

**Table:**

	*U*^11^	*U*^22^	*U*^33^	*U*^12^	*U*^13^	*U*^23^
Ag1	0.01209 (9)	0.05204 (18)	0.01674 (11)	0.000	0.00362 (8)	0.000
Ag2	0.03519 (13)	0.01557 (9)	0.01224 (9)	−0.01110 (8)	0.00786 (9)	−0.00296 (7)
Zn2	0.00905 (11)	0.01104 (12)	0.00942 (12)	0.000	0.00472 (9)	0.000
Zn1	0.00607 (8)	0.00863 (9)	0.00535 (9)	0.00074 (6)	0.00264 (6)	0.00062 (6)
Fe1	0.00607 (8)	0.00863 (9)	0.00535 (9)	0.00074 (6)	0.00264 (6)	0.00062 (6)
V1	0.00655 (10)	0.00709 (10)	0.00474 (10)	0.00040 (8)	0.00202 (8)	0.00021 (8)
V2	0.00649 (14)	0.00644 (14)	0.00448 (14)	0.000	0.00112 (11)	0.000
O1	0.0095 (5)	0.0130 (5)	0.0160 (6)	0.0017 (4)	0.0045 (5)	0.0008 (5)
O2	0.0140 (6)	0.0155 (6)	0.0082 (5)	0.0017 (5)	0.0046 (4)	−0.0004 (4)
O3	0.0149 (6)	0.0116 (5)	0.0158 (6)	−0.0010 (4)	0.0061 (5)	0.0017 (5)
O4	0.0123 (5)	0.0133 (5)	0.0078 (5)	0.0037 (4)	0.0034 (4)	0.0019 (4)
O5	0.0090 (5)	0.0135 (5)	0.0075 (5)	0.0019 (4)	0.0035 (4)	0.0027 (4)
O6	0.0087 (5)	0.0103 (5)	0.0136 (6)	−0.0007 (4)	0.0019 (4)	0.0016 (4)

### Geometric parameters (Å, º)

**Table:**

Ag1—O3^i^	2.4699 (15)	Zn2—O1^v^	2.1619 (15)
Ag1—O3^ii^	2.4699 (16)	Zn1—O6^viii^	2.0068 (14)
Ag1—O3^iii^	2.4734 (16)	Zn1—O4^x^	2.0222 (14)
Ag1—O3	2.4734 (16)	Zn1—O3^i^	2.0241 (15)
Ag2—O6^iv^	2.4374 (14)	Zn1—O2	2.0540 (14)
Ag2—O6^iii^	2.4374 (14)	Zn1—O5	2.0675 (13)
Ag2—O1^v^	2.5032 (15)	Zn1—O2^viii^	2.2082 (15)
Ag2—O1^vi^	2.5032 (15)	V1—O1	1.6784 (14)
Ag2—O1^vii^	2.5873 (14)	V1—O2	1.7343 (14)
Ag2—O1^viii^	2.5873 (14)	V1—O3	1.7372 (15)
Zn2—O5^ix^	2.0704 (14)	V1—O4	1.7402 (13)
Zn2—O5	2.0705 (14)	V2—O6	1.6984 (14)
Zn2—O4^iii^	2.1325 (13)	V2—O6^iii^	1.6984 (14)
Zn2—O4^x^	2.1325 (13)	V2—O5^iii^	1.7544 (13)
Zn2—O1^viii^	2.1619 (15)	V2—O5	1.7544 (13)
			
O3^i^—Ag1—O3^ii^	179.15 (7)	O5—Zn2—O1^v^	107.36 (5)
O3^i^—Ag1—O3^iii^	92.83 (5)	O4^iii^—Zn2—O1^v^	85.01 (5)
O3^ii^—Ag1—O3^iii^	87.10 (5)	O4^x^—Zn2—O1^v^	161.31 (5)
O3^i^—Ag1—O3	87.10 (5)	O1^viii^—Zn2—O1^v^	76.52 (7)
O3^ii^—Ag1—O3	92.83 (5)	O6^viii^—Zn1—O4^x^	104.63 (6)
O3^iii^—Ag1—O3	169.95 (7)	O6^viii^—Zn1—O3^i^	91.33 (6)
O6^iv^—Ag2—O6^iii^	180.00 (6)	O4^x^—Zn1—O3^i^	89.94 (6)
O6^iv^—Ag2—O1^v^	105.99 (5)	O6^viii^—Zn1—O2	90.95 (6)
O6^iii^—Ag2—O1^v^	74.01 (5)	O4^x^—Zn1—O2	161.09 (6)
O6^iv^—Ag2—O1^vi^	74.01 (5)	O3^i^—Zn1—O2	100.52 (6)
O6^iii^—Ag2—O1^vi^	105.99 (5)	O6^viii^—Zn1—O5	168.70 (6)
O1^v^—Ag2—O1^vi^	180.00 (6)	O4^x^—Zn1—O5	79.73 (5)
O6^iv^—Ag2—O1^vii^	107.39 (5)	O3^i^—Zn1—O5	99.16 (6)
O6^iii^—Ag2—O1^vii^	72.61 (5)	O2—Zn1—O5	83.05 (5)
O1^v^—Ag2—O1^vii^	116.56 (6)	O6^viii^—Zn1—O2^viii^	80.30 (5)
O1^vi^—Ag2—O1^vii^	63.44 (6)	O4^x^—Zn1—O2^viii^	89.43 (5)
O6^iv^—Ag2—O1^viii^	72.61 (5)	O3^i^—Zn1—O2^viii^	171.16 (6)
O6^iii^—Ag2—O1^viii^	107.39 (5)	O2—Zn1—O2^viii^	82.59 (6)
O1^v^—Ag2—O1^viii^	63.44 (6)	O5—Zn1—O2^viii^	89.40 (5)
O1^vi^—Ag2—O1^viii^	116.56 (6)	O1—V1—O2	106.24 (7)
O1^vii^—Ag2—O1^viii^	180.0	O1—V1—O3	111.93 (7)
O5^ix^—Zn2—O5	149.81 (8)	O2—V1—O3	110.32 (7)
O5^ix^—Zn2—O4^iii^	77.17 (5)	O1—V1—O4	108.88 (7)
O5—Zn2—O4^iii^	86.37 (5)	O2—V1—O4	112.54 (7)
O5^ix^—Zn2—O4^x^	86.37 (5)	O3—V1—O4	107.01 (7)
O5—Zn2—O4^x^	77.17 (5)	O6—V2—O6^iii^	102.56 (10)
O4^iii^—Zn2—O4^x^	113.56 (8)	O6—V2—O5^iii^	108.46 (7)
O5^ix^—Zn2—O1^viii^	107.36 (5)	O6^iii^—V2—O5^iii^	109.48 (6)
O5—Zn2—O1^viii^	96.35 (6)	O6—V2—O5	109.48 (6)
O4^iii^—Zn2—O1^viii^	161.31 (5)	O6^iii^—V2—O5	108.47 (7)
O4^x^—Zn2—O1^viii^	85.01 (5)	O5^iii^—V2—O5	117.36 (9)
O5^ix^—Zn2—O1^v^	96.35 (6)		

Symmetry codes: (i) *x*, −*y*+1, *z*−1/2; (ii) −*x*+1, −*y*+1, −*z*+2; (iii) −*x*+1, *y*, −*z*+3/2; (iv) *x*, −*y*, *z*−1/2; (v) *x*+1/2, −*y*+1/2, *z*−1/2; (vi) −*x*+1/2, *y*−1/2, −*z*+3/2; (vii) *x*+1/2, *y*−1/2, *z*; (viii) −*x*+1/2, −*y*+1/2, −*z*+1; (ix) −*x*+1, *y*, −*z*+1/2; (x) *x*, *y*, *z*−1.
